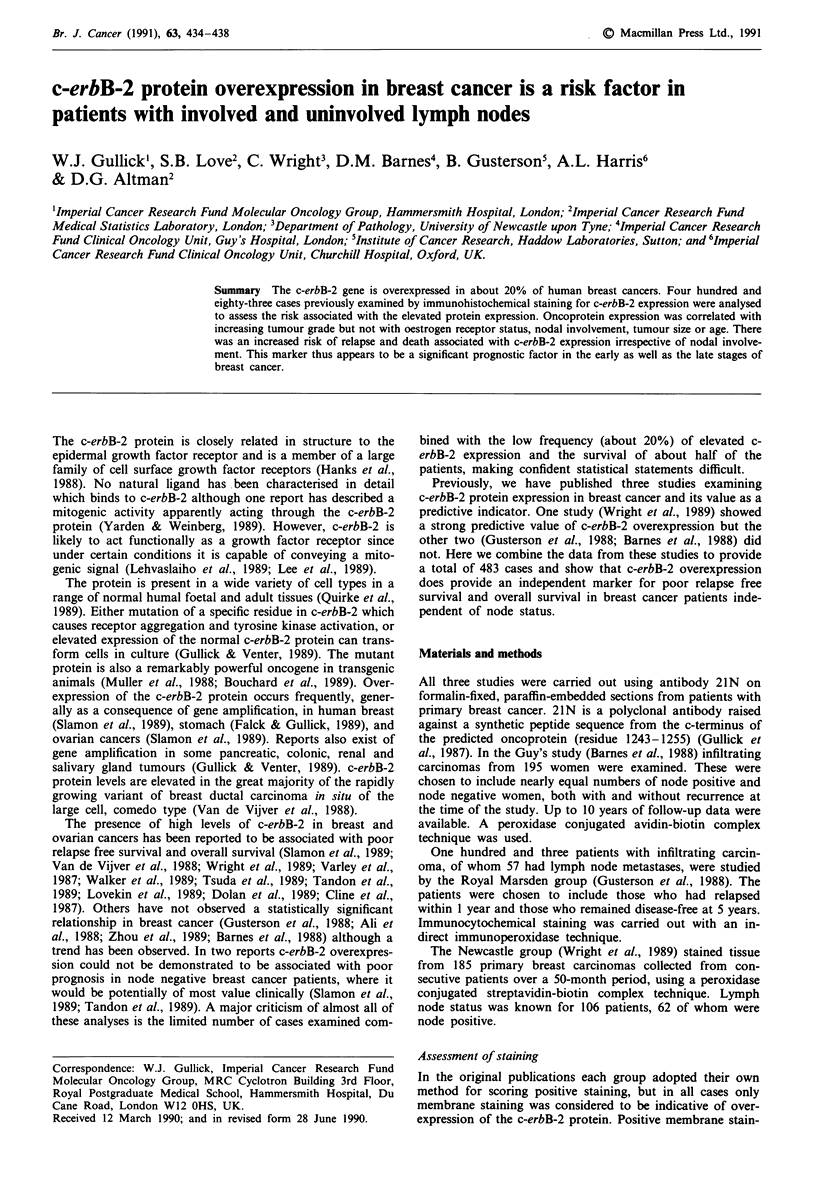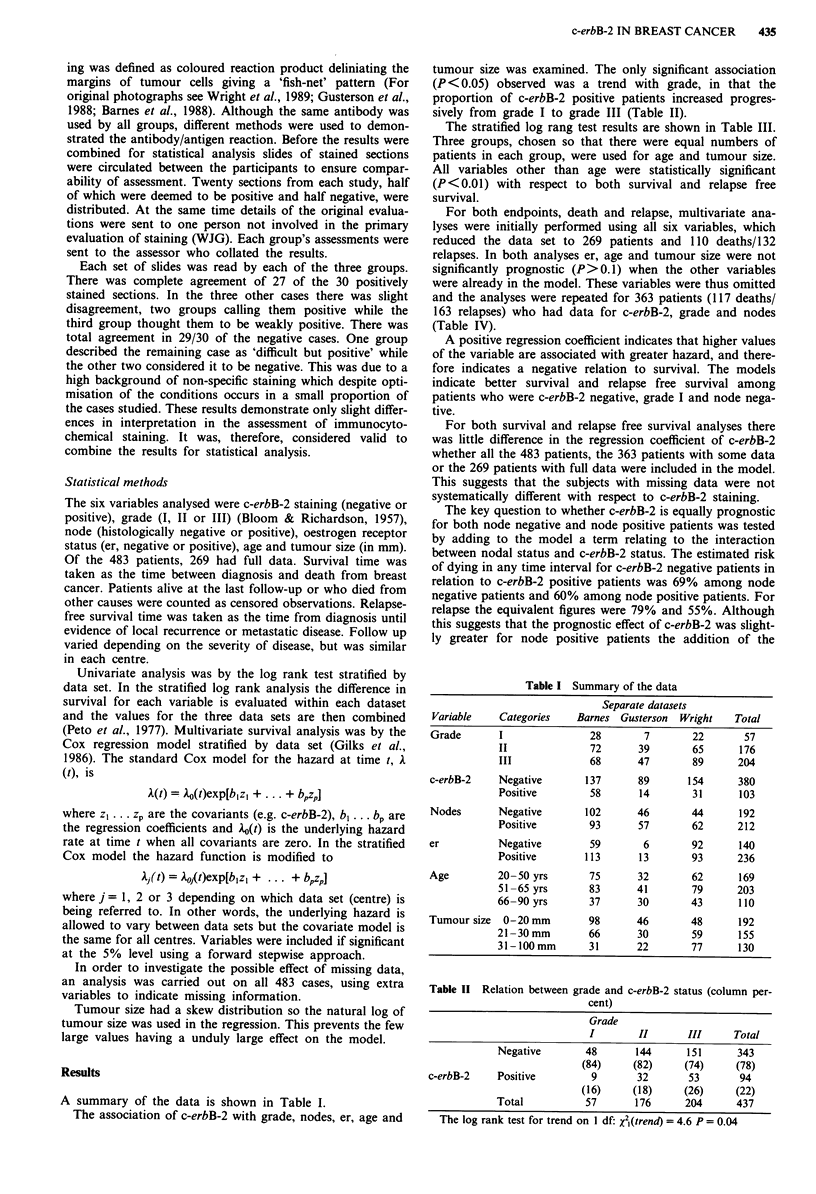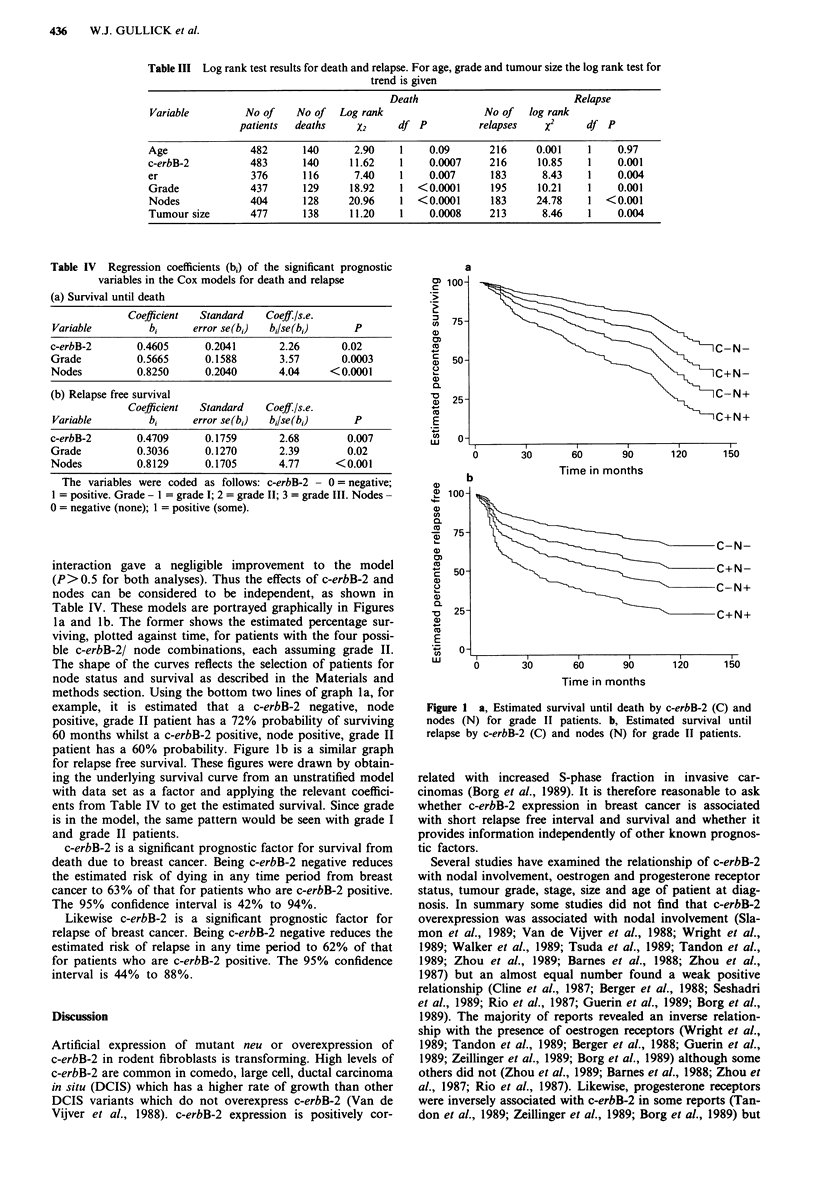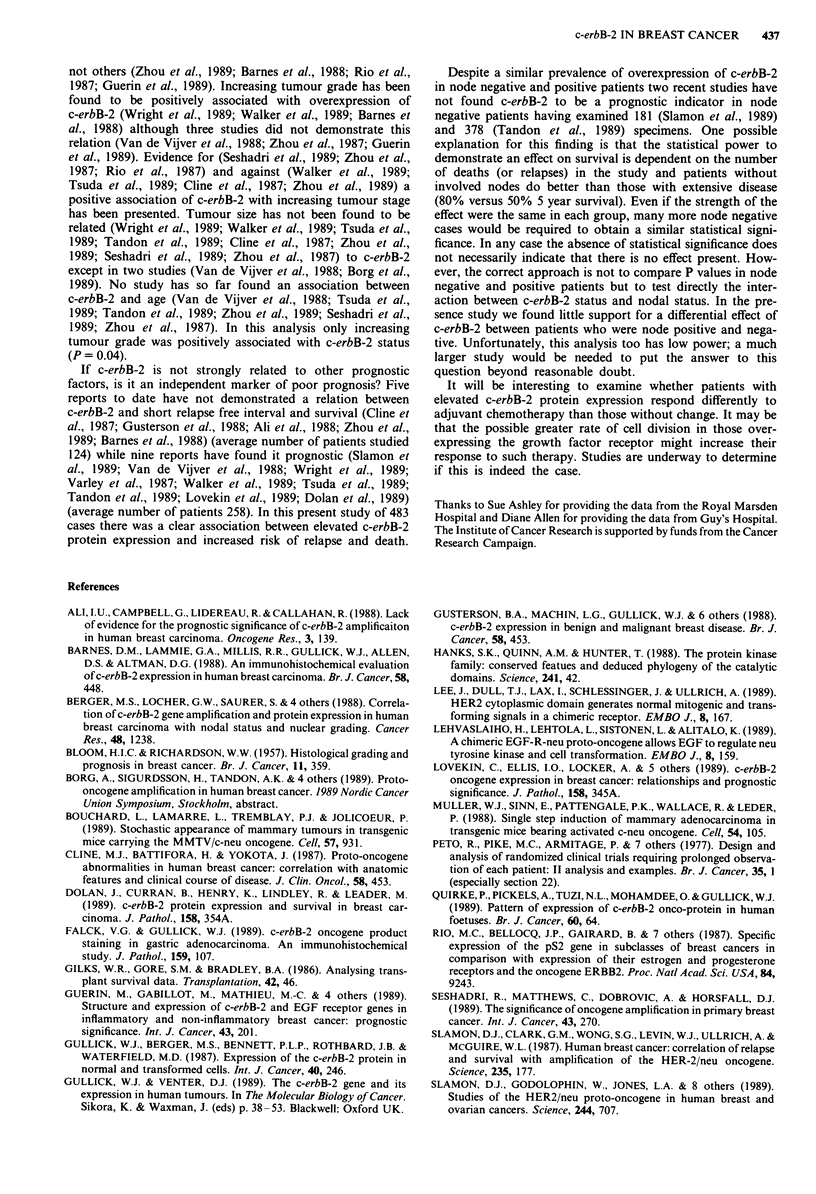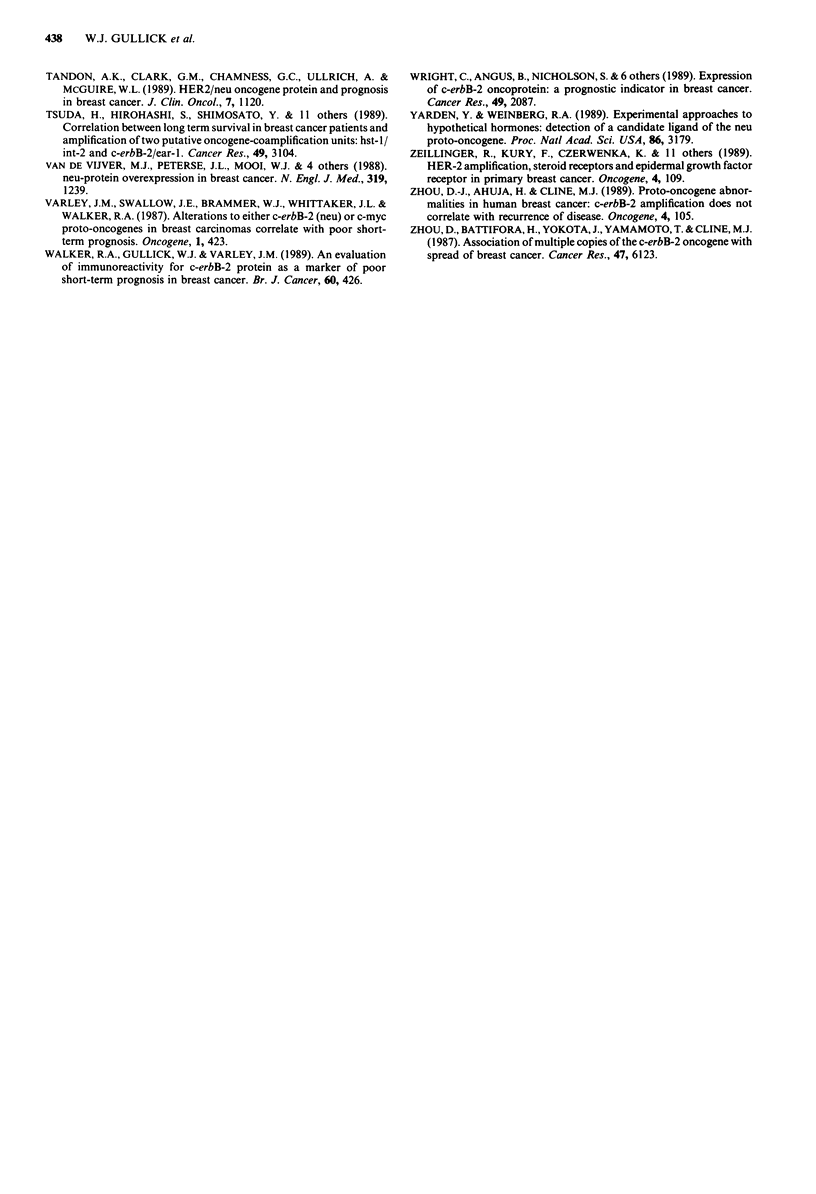# c-erbB-2 protein overexpression in breast cancer is a risk factor in patients with involved and uninvolved lymph nodes.

**DOI:** 10.1038/bjc.1991.100

**Published:** 1991-03

**Authors:** W. J. Gullick, S. B. Love, C. Wright, D. M. Barnes, B. Gusterson, A. L. Harris, D. G. Altman

**Affiliations:** Imperial Cancer Research Fund Molecular Oncology Group, Hammersmith Hospital, London, UK.

## Abstract

The c-erbB-2 gene is overexpressed in about 20% of human breast cancers. Four hundred and eighty-three cases previously examined by immunohistochemical staining for c-erbB-2 expression were analysed to assess the risk associated with the elevated protein expression. Oncoprotein expression was correlated with increasing tumour grade but not with oestrogen receptor status, nodal involvement, tumour size or age. There was an increased risk of relapse and death associated with c-erbB-2 expression irrespective of nodal involvement. This marker thus appears to be a significant prognostic factor in the early as well as the late stages of breast cancer.


*Br. J. Cancer* (1991), **63**, 434–438

© Macmillan Press Ltd., 1991

### c-*erb*B-2 protein overexpression in breast cancer is a risk factor in patients with involved and uninvolved lymph nodes

W.J. Gullick[1], S.B. Love[2], C. Wright[3], D.M. Barnes[4], B. Gusterson[5], A.L. Harris[6] & D.G. Altman[2]

[1]*Imperial Cancer Research Fund Molecular Oncology Group, Hammersmith Hospital, London;* [2]*Imperial Cancer Research Fund Medical Statistics Laboratory, London;* [3]*Department of Pathology, University of Newcastle upon Tyne;* [4]*Imperial Cancer Research Fund Clinical Oncology Unit, Guy's Hospital, London;* [5]*Institute of Cancer Research, Haddow Laboratories, Sutton; and* [6]*Imperial Cancer Research Fund Clinical Oncology Unit, Churchill Hospital, Oxford, UK.*

**Summary** The c-*erb*B-2 gene is overexpressed in about 20% of human breast cancers. Four hundred and eighty-three cases previously examined by immunohistochemical staining for c-*erb*B-2 expression were analysed to assess the risk associated with the elevated protein expression. Oncoprotein expression was correlated with increasing tumour grade but not with oestrogen receptor status, nodal involvement, tumour size or age. There was an increased risk of relapse and death associated with c-*erb*B-2 expression irrespective of nodal involvement. This marker thus appears to be a significant prognostic factor in the early as well as the late stages of breast cancer.

The c-*erb*B-2 protein is closely related in structure to the epidermal growth factor receptor and is a member of a large family of cell surface growth factor receptors (Hanks *et al.*, 1988). No natural ligand has been characterised in detail which binds to c-*erb*B-2 although one report has described a mitogenic activity apparently acting through the c-*erb*B-2 protein (Yarden & Weinberg, 1989). However, c-*erb*B-2 is likely to act functionally as a growth factor receptor since under certain conditions it is capable of conveying a mitogenic signal (Lehvaslaiho *et al.*, 1989; Lee *et al.*, 1989).

The protein is present in a wide variety of cell types in a range of normal humal foetal and adult tissues (Quirke *et al.*, 1989). Either mutation of a specific residue in c-*erb*B-2 which causes receptor aggregation and tyrosine kinase activation, or elevated expression of the normal c-*erb*B-2 protein can transform cells in culture (Gullick & Venter, 1989). The mutant protein is also a remarkably powerful oncogene in transgenic animals (Muller *et al.*, 1988; Bouchard *et al.*, 1989). Overexpression of the c-*erb*B-2 protein occurs frequently, generally as a consequence of gene amplification, in human breast (Slamon *et al.*, 1989), stomach (Falck & Gullick, 1989), and ovarian cancers (Slamon *et al.*, 1989). Reports also exist of gene amplification in some pancreatic, colonic, renal and salivary gland tumours (Gullick & Venter, 1989). c-*erb*B-2 protein levels are elevated in the great majority of the rapidly growing variant of breast ductal carcinoma *in situ* of the large cell, comedo type (Van de Vijver *et al.*, 1988).

The presence of high levels of c-*erb*B-2 in breast and ovarian cancers has been reported to be associated with poor relapse free survival and overall survival (Slamon *et al.*, 1989; Van de Vijver *et al.*, 1988; Wright *et al.*, 1989; Varley *et al.*, 1987; Walker *et al.*, 1989; Tsuda *et al.*, 1989; Tandon *et al.*, 1989; Lovekin *et al.*, 1989; Dolan *et al.*, 1989; Cline *et al.*, 1987). Others have not observed a statistically significant relationship in breast cancer (Gusterson *et al.*, 1988; Ali *et al.*, 1988; Zhou *et al.*, 1989; Barnes *et al.*, 1988) although a trend has been observed. In two reports c-*erb*B-2 overexpression could not be demonstrated to be associated with poor prognosis in node negative breast cancer patients, where it would be potentially of most value clinically (Slamon *et al.*, 1989; Tandon *et al.*, 1989). A major criticism of almost all of these analyses is the limited number of cases examined combined with the low frequency (about 20%) of elevated c-*erb*B-2 expression and the survival of about half of the patients, making confident statistical statements difficult.

Previously, we have published three studies examining c-*erb*B-2 protein expression in breast cancer and its value as a predictive indicator. One study (Wright *et al.*, 1989) showed a strong predictive value of c-*erb*B-2 overexpression but the other two (Gusterson *et al.*, 1988; Barnes *et al.*, 1988) did not. Here we combine the data from these studies to provide a total of 483 cases and show that c-*erb*B-2 overexpression does provide an independent marker for poor relapse free survival and overall survival in breast cancer patients independent of node status.

### Materials and methods

All three studies were carried out using antibody 21N on formalin-fixed, paraffin-embedded sections from patients with primary breast cancer. 21N is a polyclonal antibody raised against a synthetic peptide sequence from the c-terminus of the predicted oncoprotein (residue 1243–1255) (Gullick *et al.*, 1987). In the Guy's study (Barnes *et al.*, 1988) infiltrating carcinomas from 195 women were examined. These were chosen to include nearly equal numbers of node positive and node negative women, both with and without recurrence at the time of the study. Up to 10 years of follow-up data were available. A peroxidase conjugated avidin-biotin complex technique was used.

One hundred and three patients with infiltrating carcinoma, of whom 57 had lymph node metastases, were studied by the Royal Marsden group (Gusterson *et al.*, 1988). The patients were chosen to include those who had relapsed within 1 year and those who remained disease-free at 5 years. Immunocytochemical staining was carried out with an indirect immunoperoxidase technique.

The Newcastle group (Wright *et al.*, 1989) stained tissue from 185 primary breast carcinomas collected from consecutive patients over a 50-month period, using a peroxidase conjugated streptavidin-biotin complex technique. Lymph node status was known for 106 patients, 62 of whom were node positive.

### *Assessment of staining*

In the original publications each group adopted their own method for scoring positive staining, but in all cases only membrane staining was considered to be indicative of overexpression of the c-*erb*B-2 protein. Positive membrane stain-

Correspondence: W.J. Gullick, Imperial Cancer Research Fund Molecular Oncology Group, MRC Cyclotron Building 3rd Floor, Royal Postgraduate Medical School, Hammersmith Hospital, Du Cane Road, London W12 0HS, UK.
Received 12 March 1990; and in revised form 28 June 1990.

ing was defined as coloured reaction product deliniating the margins of tumour cells giving a 'fish-net' pattern (For original photographs see Wright *et al*., 1989; Gusterson *et al*., 1988; Barnes *et al*., 1988). Although the same antibody was used by all groups, different methods were used to demonstrated the antibody/antigen reaction. Before the results were combined for statistical analysis slides of stained sections were circulated between the participants to ensure comparability of assessment. Twenty sections from each study, half of which were deemed to be positive and half negative, were distributed. At the same time details of the original evaluations were sent to one person not involved in the primary evaluation of staining (WJG). Each group's assessments were sent to the assessor who collated the results.

Each set of slides was read by each of the three groups. There was complete agreement of 27 of the 30 positively stained sections. In the three other cases there was slight disagreement, two groups calling them positive while the third group thought them to be weakly positive. There was total agreement in 29/30 of the negative cases. One group described the remaining case as 'difficult but positive' while the other two considered it to be negative. This was due to a high background of non-specific staining which despite optimisation of the conditions occurs in a small proportion of the cases studied. These results demonstrate only slight differences in interpretation in the assessment of immunocytochemical staining. It was, therefore, considered valid to combine the results for statistical analysis.

*Statistical methods*

The six variables analysed were c-*erb*B-2 staining (negative or positive), grade (I, II or III) (Bloom & Richardson, 1957), node (histologically negative or positive), oestrogen receptor status (er, negative or positive), age and tumour size (in mm). Of the 483 patients, 269 had full data. Survival time was taken as the time between diagnosis and death from breast cancer. Patients alive at the last follow-up or who died from other causes were counted as censored observations. Relapse-free survival time was taken as the time from diagnosis until evidence of local recurrence or metastatic disease. Follow up varied depending on the severity of disease, but was similar in each centre.

Univariate analysis was by the log rank test stratified by data set. In the stratified log rank analysis the difference in survival for each variable is evaluated within each dataset and the values for the three data sets are then combined (Peto *et al*., 1977). Multivariate survival analysis was by the Cox regression model stratified by data set (Gilks *et al*., 1986). The standard Cox model for the hazard at time $t$, $\lambda(t)$, is

$$\lambda(t) = \lambda_0(t)\exp[b_1z_1 + \ldots + b_pz_p]$$

where $z_1 \ldots z_p$ are the covariants (e.g. c-*erb*B-2), $b_1 \ldots b_p$ are the regression coefficients and $\lambda_0(t)$ is the underlying hazard rate at time $t$ when all covariants are zero. In the stratified Cox model the hazard function is modified to

$$\lambda_j(t) = \lambda_{0j}(t)\exp[b_1z_1 + \ldots + b_pz_p]$$

where $j = 1$, 2 or 3 depending on which data set (centre) is being referred to. In other words, the underlying hazard is allowed to vary between data sets but the covariate model is the same for all centres. Variables were included if significant at the 5% level using a forward stepwise approach.

In order to investigate the possible effect of missing data, an analysis was carried out on all 483 cases, using extra variables to indicate missing information.

Tumour size had a skew distribution so the natural log of tumour size was used in the regression. This prevents the few large values having a unduly large effect on the model.

### Results

A summary of the data is shown in Table I.

The association of c-*erb*B-2 with grade, nodes, er, age and tumour size was examined. The only significant association ($P<0.05$) observed was a trend with grade, in that the proportion of c-*erb*B-2 positive patients increased progressively from grade I to grade III (Table II).

The stratified log rang test results are shown in Table III. Three groups, chosen so that there were equal numbers of patients in each group, were used for age and tumour size. All variables other than age were statistically significant ($P<0.01$) with respect to both survival and relapse free survival.

For both endpoints, death and relapse, multivariate analyses were initially performed using all six variables, which reduced the data set to 269 patients and 110 deaths/132 relapses. In both analyses er, age and tumour size were not significantly prognostic ($P>0.1$) when the other variables were already in the model. These variables were thus omitted and the analyses were repeated for 363 patients (117 deaths/163 relapses) who had data for c-*erb*B-2, grade and nodes (Table IV).

A positive regression coefficient indicates that higher values of the variable are associated with greater hazard, and therefore indicates a negative relation to survival. The models indicate better survival and relapse free survival among patients who were c-*erb*B-2 negative, grade I and node negative.

For both survival and relapse free survival analyses there was little difference in the regression coefficient of c-*erb*B-2 whether all the 483 patients, the 363 patients with some data or the 269 patients with full data were included in the model. This suggests that the subjects with missing data were not systematically different with respect to c-*erb*B-2 staining.

The key question to whether c-*erb*B-2 is equally prognostic for both node negative and node positive patients was tested by adding to the model a term relating to the interaction between nodal status and c-*erb*B-2 status. The estimated risk of dying in any time interval for c-*erb*B-2 negative patients in relation to c-*erb*B-2 positive patients was 69% among node negative patients and 60% among node positive patients. For relapse the equivalent figures were 79% and 55%. Although this suggests that the prognostic effect of c-*erb*B-2 was slightly greater for node positive patients the addition of the

**Table I** Summary of the data

| Variable | Categories | Separate datasets Barnes | Gusterson | Wright | Total |
|---|---|---|---|---|---|
| Grade | I | 28 | 7 | 22 | 57 |
| | II | 72 | 39 | 65 | 176 |
| | III | 68 | 47 | 89 | 204 |
| c-*erb*B-2 | Negative | 137 | 89 | 154 | 380 |
| | Positive | 58 | 14 | 31 | 103 |
| Nodes | Negative | 102 | 46 | 44 | 192 |
| | Positive | 93 | 57 | 62 | 212 |
| er | Negative | 59 | 6 | 92 | 140 |
| | Positive | 113 | 13 | 93 | 236 |
| Age | 20–50 yrs | 75 | 32 | 62 | 169 |
| | 51–65 yrs | 83 | 41 | 79 | 203 |
| | 66–90 yrs | 37 | 30 | 43 | 110 |
| Tumour size | 0–20 mm | 98 | 46 | 48 | 192 |
| | 21–30 mm | 66 | 30 | 59 | 155 |
| | 31–100 mm | 31 | 22 | 77 | 130 |

**Table II** Relation between grade and c-*erb*B-2 status (column percent)

| | | Grade I | II | III | Total |
|---|---|---|---|---|---|
| | Negative | 48 | 144 | 151 | 343 |
| | | (84) | (82) | (74) | (78) |
| c-*erb*B-2 | Positive | 9 | 32 | 53 | 94 |
| | | (16) | (18) | (26) | (22) |
| | Total | 57 | 176 | 204 | 437 |

The log rank test for trend on 1 df: $\chi^2_1(trend) = 4.6$ $P = 0.04$

**Table III** Log rank test results for death and relapse. For age, grade and tumour size the log rank test for trend is given

| | Death | | | | | Relapse | | | |
|---|---|---|---|---|---|---|---|---|---|
| *Variable* | *No of patients* | *No of deaths* | *Log rank* $\chi_2$ | *df* | *P* | *No of relapses* | *log rank* $\chi^2$ | *df* | *P* |
| Age | 482 | 140 | 2.90 | 1 | 0.09 | 216 | 0.001 | 1 | 0.97 |
| c-*erb*B-2 | 483 | 140 | 11.62 | 1 | 0.0007 | 216 | 10.85 | 1 | 0.001 |
| er | 376 | 116 | 7.40 | 1 | 0.007 | 183 | 8.43 | 1 | 0.004 |
| Grade | 437 | 129 | 18.92 | 1 | <0.0001 | 195 | 10.21 | 1 | 0.001 |
| Nodes | 404 | 128 | 20.96 | 1 | <0.0001 | 183 | 24.78 | 1 | <0.001 |
| Tumour size | 477 | 138 | 11.20 | 1 | 0.0008 | 213 | 8.46 | 1 | 0.004 |

**Table IV** Regression coefficients ($b_i$) of the significant prognostic variables in the Cox models for death and relapse

(a) Survival until death

| *Variable* | *Coefficient* $b_i$ | *Standard error* $se(b_i)$ | *Coeff./s.e.* $b_i/se(b_i)$ | *P* |
|---|---|---|---|---|
| c-*erb*B-2 | 0.4605 | 0.2041 | 2.26 | 0.02 |
| Grade | 0.5665 | 0.1588 | 3.57 | 0.0003 |
| Nodes | 0.8250 | 0.2040 | 4.04 | <0.0001 |

(b) Relapse free survival

| *Variable* | *Coefficient* $b_i$ | *Standard error* $se(b_i)$ | *Coeff./s.e.* $b_i/se(b_i)$ | *P* |
|---|---|---|---|---|
| c-*erb*B-2 | 0.4709 | 0.1759 | 2.68 | 0.007 |
| Grade | 0.3036 | 0.1270 | 2.39 | 0.02 |
| Nodes | 0.8129 | 0.1705 | 4.77 | <0.001 |

The variables were coded as follows: c-*erb*B-2 – 0 = negative; 1 = positive. Grade – 1 = grade I; 2 = grade II; 3 = grade III. Nodes – 0 = negative (none); 1 = positive (some).

**Figure 1** **a**, Estimated survival until death by c-*erb*B-2 (C) and nodes (N) for grade II patients. **b**, Estimated survival until relapse by c-*erb*B-2 (C) and nodes (N) for grade II patients.

interaction gave a negligible improvement to the model ($P > 0.5$ for both analyses). Thus the effects of c-*erb*B-2 and nodes can be considered to be independent, as shown in Table IV. These models are portrayed graphically in Figures 1a and 1b. The former shows the estimated percentage surviving, plotted against time, for patients with the four possible c-*erb*B-2/ node combinations, each assuming grade II. The shape of the curves reflects the selection of patients for node status and survival as described in the Materials and methods section. Using the bottom two lines of graph 1a, for example, it is estimated that a c-*erb*B-2 negative, node positive, grade II patient has a 72% probability of surviving 60 months whilst a c-*erb*B-2 positive, node positive, grade II patient has a 60% probability. Figure 1b is a similar graph for relapse free survival. These figures were drawn by obtaining the underlying survival curve from an unstratified model with data set as a factor and applying the relevant coefficients from Table IV to get the estimated survival. Since grade is in the model, the same pattern would be seen with grade I and grade II patients.

c-*erb*B-2 is a significant prognostic factor for survival from death due to breast cancer. Being c-*erb*B-2 negative reduces the estimated risk of dying in any time period from breast cancer to 63% of that for patients who are c-*erb*B-2 positive. The 95% confidence interval is 42% to 94%.

Likewise c-*erb*B-2 is a significant prognostic factor for relapse of breast cancer. Being c-*erb*B-2 negative reduces the estimated risk of relapse in any time period to 62% of that for patients who are c-*erb*B-2 positive. The 95% confidence interval is 44% to 88%.

### Discussion

Artificial expression of mutant *neu* or overexpression of c-*erb*B-2 in rodent fibroblasts is transforming. High levels of c-*erb*B-2 are common in comedo, large cell, ductal carcinoma *in situ* (DCIS) which has a higher rate of growth than other DCIS variants which do not overexpress c-*erb*B-2 (Van de Vijver *et al.*, 1988). c-*erb*B-2 expression is positively correlated with increased S-phase fraction in invasive carcinomas (Borg *et al.*, 1989). It is therefore reasonable to ask whether c-*erb*B-2 expression in breast cancer is associated with short relapse free interval and survival and whether it provides information independently of other known prognostic factors.

Several studies have examined the relationship of c-*erb*B-2 with nodal involvement, oestrogen and progesterone receptor status, tumour grade, stage, size and age of patient at diagnosis. In summary some studies did not find that c-*erb*B-2 overexpression was associated with nodal involvement (Slamon *et al.*, 1989; Van de Vijver *et al.*, 1988; Wright *et al.*, 1989; Walker *et al.*, 1989; Tsuda *et al.*, 1989; Tandon *et al.*, 1989; Zhou *et al.*, 1989; Barnes *et al.*, 1988; Zhou *et al.*, 1987) but an almost equal number found a weak positive relationship (Cline *et al.*, 1987; Berger *et al.*, 1988; Seshadri *et al.*, 1989; Rio *et al.*, 1987; Guerin *et al.*, 1989; Borg *et al.*, 1989). The majority of reports revealed an inverse relationship with the presence of oestrogen receptors (Wright *et al.*, 1989; Tandon *et al.*, 1989; Berger *et al.*, 1988; Guerin *et al.*, 1989; Zeillinger *et al.*, 1989; Borg *et al.*, 1989) although some others did not (Zhou *et al.*, 1989; Barnes *et al.*, 1988; Zhou *et al.*, 1987; Rio *et al.*, 1987). Likewise, progesterone receptors were inversely associated with c-*erb*B-2 in some reports (Tandon *et al.*, 1989; Zeillinger *et al.*, 1989; Borg *et al.*, 1989) but not others (Zhou *et al.*, 1989; Barnes *et al.*, 1988; Rio *et al.*, 1987; Guerin *et al.*, 1989). Increasing tumour grade has been found to be positively associated with overexpression of c-*erb*B-2 (Wright *et al.*, 1989; Walker *et al.*, 1989; Barnes *et al.*, 1988) although three studies did not demonstrate this relation (Van de Vijver *et al.*, 1988; Zhou *et al.*, 1987; Guerin *et al.*, 1989). Evidence for (Seshadri *et al.*, 1989; Zhou *et al.*, 1987; Rio *et al.*, 1987) and against (Walker *et al.*, 1989; Tsuda *et al.*, 1989; Cline *et al.*, 1987; Zhou *et al.*, 1989) a positive association of c-*erb*B-2 with increasing tumour stage has been presented. Tumour size has not been found to be related (Wright *et al.*, 1989; Walker *et al.*, 1989; Tsuda *et al.*, 1989; Tandon *et al.*, 1989; Cline *et al.*, 1987; Zhou *et al.*, 1989; Seshadri *et al.*, 1989; Zhou *et al.*, 1987) to c-*erb*B-2 except in two studies (Van de Vijver *et al.*, 1988; Borg *et al.*, 1989). No study has so far found an association between c-*erb*B-2 and age (Van de Vijver *et al.*, 1988; Tsuda *et al.*, 1989; Tandon *et al.*, 1989; Zhou *et al.*, 1989; Seshadri *et al.*, 1989; Zhou *et al.*, 1987). In this analysis only increasing tumour grade was positively associated with c-*erb*B-2 status ($P = 0.04$).

If c-*erb*B-2 is not strongly related to other prognostic factors, is it an independent marker of poor prognosis? Five reports to date have not demonstrated a relation between c-*erb*B-2 and short relapse free interval and survival (Cline *et al.*, 1987; Gusterson *et al.*, 1988; Ali *et al.*, 1988; Zhou *et al.*, 1989; Barnes *et al.*, 1988) (average number of patients studied 124) while nine reports have found it prognostic (Slamon *et al.*, 1989; Van de Vijver *et al.*, 1988; Wright *et al.*, 1989; Varley *et al.*, 1987; Walker *et al.*, 1989; Tsuda *et al.*, 1989; Tandon *et al.*, 1989; Lovekin *et al.*, 1989; Dolan *et al.*, 1989) (average number of patients 258). In this present study of 483 cases there was a clear association between elevated c-*erb*B-2 protein expression and increased risk of relapse and death.

Despite a similar prevalence of overexpression of c-*erb*B-2 in node negative and positive patients two recent studies have not found c-*erb*B-2 to be a prognostic indicator in node negative patients having examined 181 (Slamon *et al.*, 1989) and 378 (Tandon *et al.*, 1989) specimens. One possible explanation for this finding is that the statistical power to demonstrate an effect on survival is dependent on the number of deaths (or relapses) in the study and patients without involved nodes do better than those with extensive disease (80% versus 50% 5 year survival). Even if the strength of the effect were the same in each group, many more node negative cases would be required to obtain a similar statistical significance. In any case the absence of statistical significance does not necessarily indicate that there is no effect present. However, the correct approach is not to compare P values in node negative and positive patients but to test directly the interaction between c-*erb*B-2 status and nodal status. In the presence study we found little support for a differential effect of c-*erb*B-2 between patients who were node positive and negative. Unfortunately, this analysis too has low power; a much larger study would be needed to put the answer to this question beyond reasonable doubt.

It will be interesting to examine whether patients with elevated c-*erb*B-2 protein expression respond differently to adjuvant chemotherapy than those without change. It may be that the possible greater rate of cell division in those overexpressing the growth factor receptor might increase their response to such therapy. Studies are underway to determine if this is indeed the case.

Thanks to Sue Ashley for providing the data from the Royal Marsden Hospital and Diane Allen for providing the data from Guy's Hospital. The Institute of Cancer Research is supported by funds from the Cancer Research Campaign.

### References

ALI, I.U., CAMPBELL, G., LIDEREAU, R. & CALLAHAN, R. (1988). Lack of evidence for the prognostic significance of c-*erb*B-2 amplificaiton in human breast carcinoma. *Oncogene Res.*, **3**, 139.

BARNES, D.M., LAMMIE, G.A., MILLIS, R.R., GULLICK, W.J., ALLEN, D.S. & ALTMAN, D.G. (1988). An immunohistochemical evaluation of c-*erb*B-2 expression in human breast carcinoma. *Br. J. Cancer*, **58**, 448.

BERGER, M.S., LOCHER, G.W., SAURER, S. & 4 others (1988). Correlation of c-*erb*B-2 gene amplification and protein expression in human breast carcinoma with nodal status and nuclear grading. *Cancer Res.*, **48**, 1238.

BLOOM, H.I.C. & RICHARDSON, W.W. (1957). Histological grading and prognosis in breast cancer. *Br. J. Cancer*, **11**, 359.

BORG, A., SIGURDSSON, H., TANDON, A.K. & 4 others (1989). Proto-oncogene amplification in human breast cancer. *1989 Nordic Cancer Union Symposium, Stockholm*, abstract.

BOUCHARD, L., LAMARRE, L., TREMBLAY, P.J. & JOLICOEUR, P. (1989). Stochastic appearance of mammary tumours in transgenic mice carrying the MMTV/c-neu oncogene. *Cell*, **57**, 931.

CLINE, M.J., BATTIFORA, H. & YOKOTA, J. (1987). Proto-oncogene abnormalities in human breast cancer: correlation with anatomic features and clinical course of disease. *J. Clin. Oncol.*, **58**, 453.

DOLAN, J., CURRAN, B., HENRY, K., LINDLEY, R. & LEADER, M. (1989). c-*erb*B-2 protein expression and survival in breast carcinoma. *J. Pathol.*, **158**, 354A.

FALCK, V.G. & GULLICK, W.J. (1989). c-*erb*B-2 oncogene product staining in gastric adenocarcinoma. An immunohistochemical study. *J. Pathol.*, **159**, 107.

GILKS, W.R., GORE, S.M. & BRADLEY, B.A. (1986). Analysing transplant survival data. *Transplantation*, **42**, 46.

GUERIN, M., GABILLOT, M., MATHIEU, M.-C. & 4 others (1989). Structure and expression of c-*erb*B-2 and EGF receptor genes in inflammatory and non-inflammatory breast cancer: prognostic significance. *Int. J. Cancer*, **43**, 201.

GULLICK, W.J., BERGER, M.S., BENNETT, P.L.P., ROTHBARD, J.B. & WATERFIELD, M.D. (1987). Expression of the c-*erb*B-2 protein in normal and transformed cells. *Int. J. Cancer*, **40**, 246.

GULLICK, W.J. & VENTER, D.J. (1989). The c-*erb*B-2 gene and its expression in human tumours. In *The Molecular Biology of Cancer*. Sikora, K. & Waxman, J. (eds) p. 38–53. Blackwell: Oxford UK.

GUSTERSON, B.A., MACHIN, L.G., GULLICK, W.J. & 6 others (1988). c-*erb*B-2 expression in benign and malignant breast disease. *Br. J. Cancer*, **58**, 453.

HANKS, S.K., QUINN, A.M. & HUNTER, T. (1988). The protein kinase family: conserved featues and deduced phylogeny of the catalytic domains. *Science*, **241**, 42.

LEE, J., DULL, T.J., LAX, I., SCHLESSINGER, J. & ULLRICH, A. (1989). HER2 cytoplasmic domain generates normal mitogenic and transforming signals in a chimeric receptor. *EMBO J.*, **8**, 167.

LEHVASLAIHO, H., LEHTOLA, L., SISTONEN, L. & ALITALO, K. (1989). A chimeric EGF-R-neu proto-oncogene allows EGF to regulate neu tyrosine kinase and cell transformation. *EMBO J.*, **8**, 159.

LOVEKIN, C., ELLIS, I.O., LOCKER, A. & 5 others (1989). c-*erb*B-2 oncogene expression in breast cancer: relationships and prognostic significance. *J. Pathol.*, **158**, 345A.

MULLER, W.J., SINN, E., PATTENGALE, P.K., WALLACE, R. & LEDER, P. (1988). Single step induction of mammary adenocarcinoma in transgenic mice bearing activated c-neu oncogene. *Cell*, **54**, 105.

PETO, R., PIKE, M.C., ARMITAGE, P. & 7 others (1977). Design and analysis of randomized clinical trials requiring prolonged observation of each patient: II analysis and examples. *Br. J. Cancer*, **35**, 1 (especially section 22).

QUIRKE, P., PICKELS, A., TUZI, N.L., MOHAMDEE, O. & GULLICK, W.J. (1989). Pattern of expression of c-*erb*B-2 onco-protein in human foetuses. *Br. J. Cancer*, **60**, 64.

RIO, M.C., BELLOCQ, J.P., GAIRARD, B. & 7 others (1987). Specific expression of the pS2 gene in subclasses of breast cancers in comparison with expression of their estrogen and progesterone receptors and the oncogene ERBB2. *Proc. Natl Acad. Sci. USA*, **84**, 9243.

SESHADRI, R., MATTHEWS, C., DOBROVIC, A. & HORSFALL, D.J. (1989). The significance of oncogene amplification in primary breast cancer. *Int. J. Cancer*, **43**, 270.

SLAMON, D.J., CLARK, G.M., WONG, S.G., LEVIN, W.J., ULLRICH, A. & MCGUIRE, W.L. (1987). Human breast cancer: correlation of relapse and survival with amplification of the HER-2/neu oncogene. *Science*, **235**, 177.

SLAMON, D.J., GODOLOPHIN, W., JONES, L.A. & 8 others (1989). Studies of the HER2/neu proto-oncogene in human breast and ovarian cancers. *Science*, **244**, 707.

TANDON, A.K., CLARK, G.M., CHAMNESS, G.C., ULLRICH, A. & MCGUIRE, W.L. (1989). HER2/neu oncogene protein and prognosis in breast cancer. *J. Clin. Oncol.*, **7**, 1120.

TSUDA, H., HIROHASHI, S., SHIMOSATO, Y. & 11 others (1989). Correlation between long term survival in breast cancer patients and amplification of two putative oncogene-coamplification units: hst-1/int-2 and c-*erb*B-2/ear-1. *Cancer Res.*, **49**, 3104.

VAN DE VIJVER, M.J., PETERSE, J.L., MOOI, W.J. & 4 others (1988). neu-protein overexpression in breast cancer. *N. Engl. J. Med.*, **319**, 1239.

VARLEY, J.M., SWALLOW, J.E., BRAMMER, W.J., WHITTAKER, J.L. & WALKER, R.A. (1987). Alterations to either c-*erb*B-2 (neu) or c-myc proto-oncogenes in breast carcinomas correlate with poor short-term prognosis. *Oncogene*, **1**, 423.

WALKER, R.A., GULLICK, W.J. & VARLEY, J.M. (1989). An evaluation of immunoreactivity for c-*erb*B-2 protein as a marker of poor short-term prognosis in breast cancer. *Br. J. Cancer*, **60**, 426.

WRIGHT, C., ANGUS, B., NICHOLSON, S. & 6 others (1989). Expression of c-*erb*B-2 oncoprotein: a prognostic indicator in breast cancer. *Cancer Res.*, **49**, 2087.

YARDEN, Y. & WEINBERG, R.A. (1989). Experimental approaches to hypothetical hormones: detection of a candidate ligand of the neu proto-oncogene. *Proc. Natl Acad. Sci. USA*, **86**, 3179.

ZEILLINGER, R., KURY, F., CZERWENKA, K. & 11 others (1989). HER-2 amplification, steroid receptors and epidermal growth factor receptor in primary breast cancer. *Oncogene*, **4**, 109.

ZHOU, D.-J., AHUJA, H. & CLINE, M.J. (1989). Proto-oncogene abnormalities in human breast cancer: c-*erb*B-2 amplification does not correlate with recurrence of disease. *Oncogene*, **4**, 105.

ZHOU, D., BATTIFORA, H., YOKOTA, J., YAMAMOTO, T. & CLINE, M.J. (1987). Association of multiple copies of the c-*erb*B-2 oncogene with spread of breast cancer. *Cancer Res.*, **47**, 6123.